# Supplementary material for: Inferring Ancestral Recombination Graphs from Bacterial Genomic Data
Source: Genetics. 2016 Dec 20;205(2):857–70. doi: 10.1534/genetics.116.193425 (PMC5289856; doi:10.1534/genetics.116.193425)
Supplement: Supplementary file 3 [file 857FileS1.pdf]

Supporting material for “Bayesian inference of ancestral recombination graphs for bacterial populations.”

File S1

1 Comparison with earlier method

The method described in this paper involves joint Bayesian inference of the full ancestral recombination graph under the ClonalOrigin model. This is in contrast with the method described by Didelot *et al.* (2010) which involves a two-step approach in which the clonal frame is first estimated separately under a separate model (Didelot and Falush, 2007) and then used as the basis of conditional inference of the rest of the recombination graph under the ClonalOrigin model.

To demonstrate the potential benifits of the joint approach in which *all* inference is done under the ClonalOrigin model, we followed the following procedure:

1. We simulated 45 ARGs under the ClonalOrigin model, each with 20 leaves and representing the ancestry of an $L = 10^4$ bp locus. (This length is typical of contigs in genomic datasets.) Simulations were performed using an expected tract length $\delta = 500$bp, a per-locus recombination rate $\rho_G = 2\rho(L + \delta - 1) = 10$ (ρ is the per-site recombination rate parameter used in this paper), and an effective population size $N = 1$. (This population size implies the time-scale is in coalescent units.)

2. Sequences were simulated down each of these ARGs under a Jukes-Cantor substitution model producing a total of 45 sequence alignments. Of these, 9 were produced under each of the following 5 values of the per-locus mutation rate $\theta_G = 2\theta L$: 3, 10, 30, 100, and 300 (θ is the per-site mutation rate parameter used in this paper).

3. Using the simulated alignments, we then inferred ARGs using both the joint method described in this paper, Bacter, and the two-step method described by Didelot *et al.* (2010), ClonalFrame+ClonalOrigin. In both cases, all parameter values were fixed to their known true values. All phases of the analyses (Bacter, ClonalFrame and ClonalOrigin) were assessed for convergence.

4. For each combination of data-set and method, the resulting ensemble of sampled ARGs was compared with the known true ARG by counting

(a) the number of true clonal frame clades which appeared in $> 50\%$ of the sampled clonal frames, and (b) the number of true recombinant edges which appeared in $> 50\%$ of the sampled ARGs. (A sampled ARG was said to contain a particular true converted edge if the ARG contained a conversion between the same pair of clades in the clonal frame as the true edge, and if the boundaries of the site region affected by conversion were within 25% of the truth relative to the length of the true affected region.)

These parameter values are in the regime where the sequence length L is much greater than the expected tract length, which is typical of larger data sets. The chosen recombination rate is much slower than that of rapidly recombining bacteria such as *Campylobacter jejuni*, which are believed to recombine at at least an order of magnitude faster than this (Wilson *et al.*, 2009).

While the ARGs are all generated from the same distribution, the mutation rate values span two orders of magnitude, yielding alignments which range from almost no diversity to having as much as 10% of their sites polymorphic (Figure S1(e)).

Figures S1(a) through S1(d) compare the inference results of the two methods. Figure S1(a) shows the percentage of clades in the true clonal frame recovered (i.e. having at least 50% posterior support) by both methods for each dataset as a function of mutation rate. While both methods suffer due to the lack of phylogenetic signal for small θ_G, the joint method displays a consistent improvement over the ClonalFrame+ClonalOrigin method (or rather the ClonalFrame method which is used for clonal frame inference). This is illustrated further in Figure S1(b), which displays the ratios of the Bacter clade recovery success fractions to the ClonalFrame+ClonalOrigin fractions. In the clear majority of cases, the joint method is superior. (Note that the ratios for the smallest θ_G value are omitted, as these are dominated by statistical noise.)

Figure S1(c) compares the percentage of recombinant edges successfully recovered (having $\geq 50\%$ posterior support) using each method. Again, both methods suffer at small θ_G values, but the joint method remains capable of recovering a significantly larger proportion of the true conversions than the ClonalFrame+ClonalOrigin method when applied to the low-diversity data sets. This is reflected also in Figure S1(d) which shows the ratios of the Bacter conversion recovery success to those of the two-step method. In all cases, the Bacter approach performs recovers on average at least twice as many conversions as ClonalFrame+ClonalOrigin, but we find that the average ratio increases to as high as 4 and 6 for smaller mutation rates. (Again, the ratios for the smallest mutation rate is omitted.)

Given the reliance of the two-step method on a point estimate of the clonal frame, it is perhaps unsurprising that it is outperformed by the joint

method when sequence diversity is low. By relying on a point estimate of the clonal frame, the posterior distribution over ARGs produced by the ClonalFrame+ClonalOrigin method must be very different to the true joint posterior when phylogenetic uncertainty is significant, as it is in the small mutation rate regime.

However, the improvement displayed by the joint method when the mutation rate requires a different explanation. This may be found by remembering that the ClonalFrame method of (Didelot and Falush, 2007) method used to produce the clonal frame estimates in the two-step method is a much stronger approximation to the coalescent with homologous gene conversion model than the ClonalOrigin model. For instance, while that model certainly accounts for recombination, it forbids conversions from altering the marginal tree topology. Furthermore, it assumes that conversions only ever act to increase sequence diversity and never to decrease it. Thus, when the signal for the clonal frame is impaired by the accumulation of recombinations, the estimate produced by ClonalFrame will suffer regardless of the strength of the phylogenetic signal due to model mispecification.

A typical example of this problem is shown in Figure S2, which compares the true clonal frame one of the $\theta_G = 300$ simulated alignments (Figure S2(a)) with a consensus tree representation of the marginal clonal frame posterior produced by the joint method (Figure S2(b)) and the consensus tree produced by the ClonalFrame phase of the joint method (Figure S2(c)). While younger features of the true clonal frame are recovered faithfully in the ClonalFrame estimate, the older coalescence ages display strong negative biased due to the build-up of recombination deeper in the genealogy. In contrast, the estimate produced by the joint method performs much better, as it is inferred under a model that properly accounts for the topological noise introduced the recombination events.

References

Didelot, X. and D. Falush, 2007 Inference of bacterial microevolution using multilocus sequence data. Genetics **175**: 1251.

Didelot, X., D. Lawson, A. Daarling, and D. Falush, 2010 Inference of homologous recombination in bacteria using whole-genome sequences. Genetics **186**: 1435.

Wilson, D. J., E. Gabriel, A. J. H. Leatherbarrow, J. Cheesbrough, S. Gee, E. Bolton, A. Fox, C. A. Hart, P. J. Diggle, and P. Fearnhead, 2009 Rapid evolution and the importance of recombination to the gastroenteric pathogen campylobacter jejuni. Mol Biol Evol **26**: 385–397.

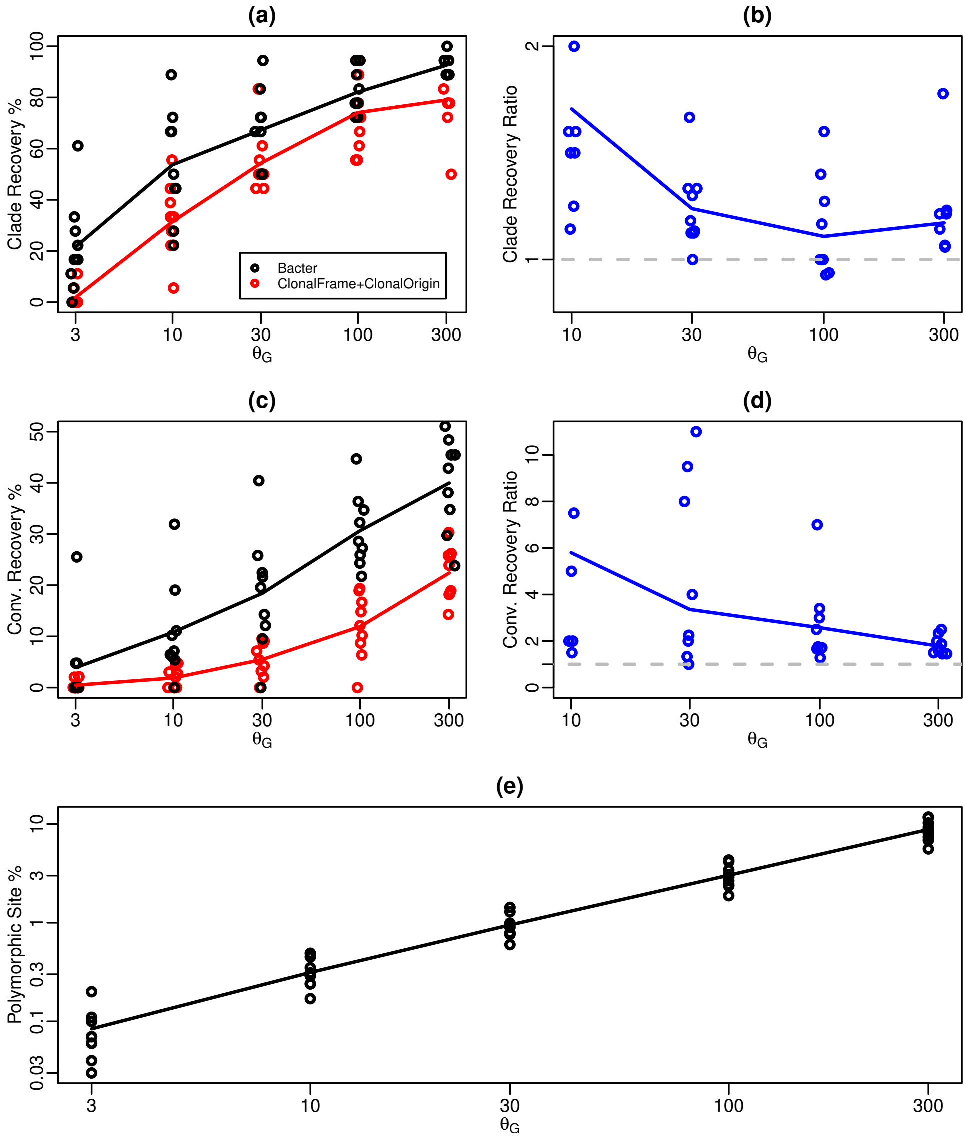

Figure S1: Comparison between capacity of our joint inference method (Bacter) and the earlier two-step method (ClonalFrame+ClonalOrigin) to recover features of ARGs from 45 simulated data sets, 9 for each of 5 distinct values of the per-locus mutation rate θ_G. (a) Comparison between percentages of recovered clonal frame clades with lines representing means. (b) Ratio of number of clades recovered by Bacter to number recovered by ClonalFrame+ClonalOrigin. (c) Comparison between percentage of true conversions recovered by each method. (d) Ratio of number of conversions recovered by Bacter to number recovered by ClonalFrame+ClonalOrigin. (e) Polymorphic site fraction for all simulated alignments as a function of mutation rate θ_G, with the line connecting the means.

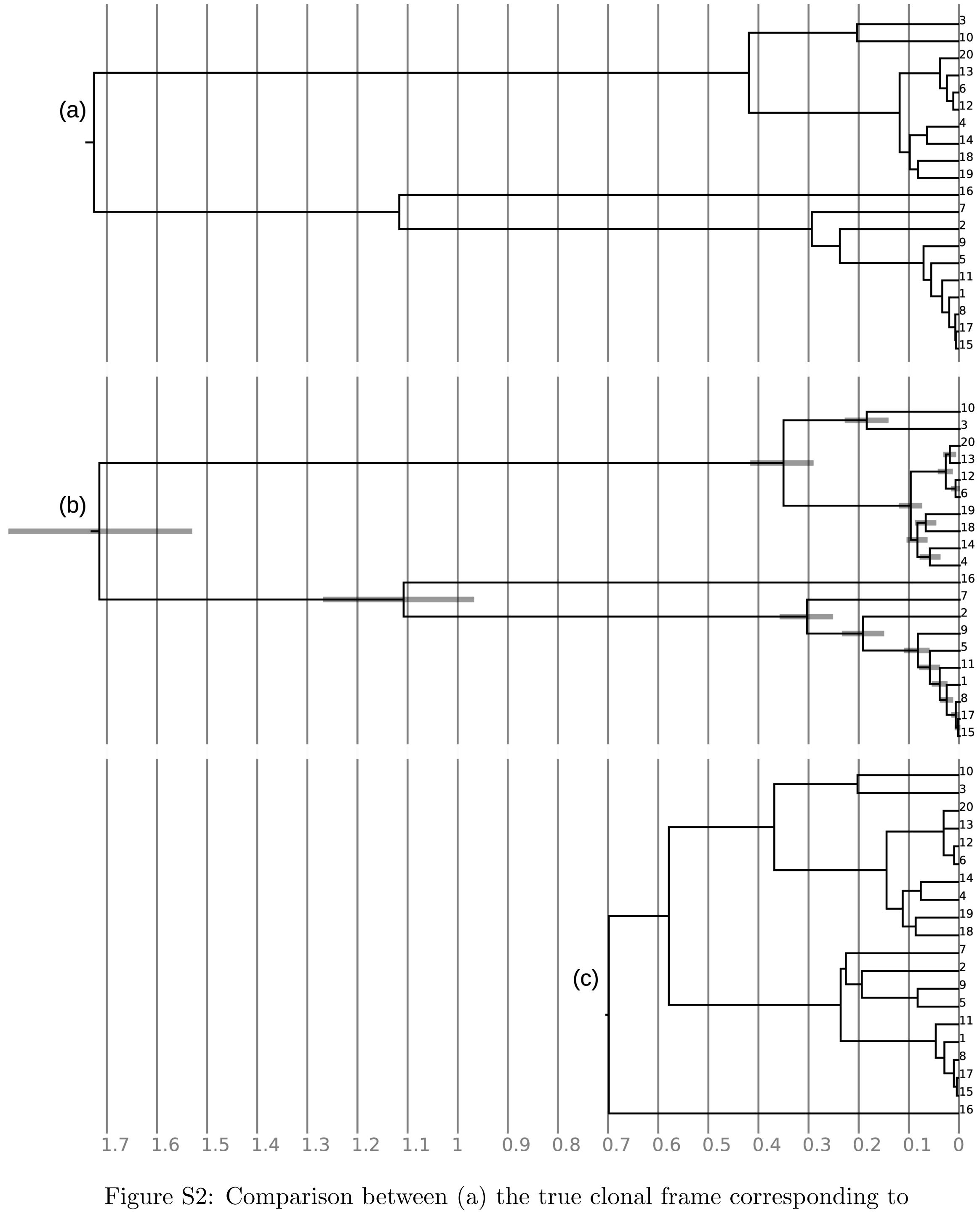

Figure S2: Comparison between (a) the true clonal frame corresponding to a single alignment simulated under the high $\theta_G = 300$ mutation rate, (b) a consensus tree produced using the joint inference method (Bacter) and (c) the consensus tree produced using the ClonalFrame method. Error bars in (b) represent 95% HPD intervals for clade ages.
