# Supplementary material for: Inferring Ancestral Recombination Graphs from Bacterial Genomic Data
Source: Genetics. 2016 Dec 20;205(2):857–70. doi: 10.1534/genetics.116.193425 (PMC5289856; doi:10.1534/genetics.116.193425)
Supplement: Supplementary file 4 [file 857FileS2.docx]

File S2. BEAST 2 analysis files. (.zip, 29 KB)

[www.genetics.org/lookup/suppl/doi:10.1534/genetics.116.193425/-/DC1/FileS2.zip](http://www.genetics.org/lookup/suppl/doi:10.1534/genetics.116.193425/-/DC1/FileS2.zip)
